# Occupational Stress and Personality in Medical Doctors from Romania

**DOI:** 10.3390/healthcare10091612

**Published:** 2022-08-24

**Authors:** Lorena Mihaela Muntean, Aurel Nireștean, Marius Mărușteri, Andreea Sima-Comaniciu, Emese Lukacs

**Affiliations:** 1 Department of Psychiatry, George Emil Palade University of Medicine, Pharmacy, Science, and Technology of Targu Mures, 540136 Targu Mures, Romania; 2 Department of Medical Informatics and Biostatistics, George Emil Palade University of Medicine, Pharmacy, Science, and Technology of Targu Mures, 540136 Targu Mures, Romania

**Keywords:** occupational stress, physician, work-life balance, work relationship, overload, personality

## Abstract

Occupational stress amongst doctors has been intensively studied as doctors are exposed to several stress factors daily. The purpose of this study was to investigate if there are associations between personality dimensions and the factors that generate stress at work. We conducted a cross-sectional study of 280 medical doctors from Romania between February 2021 and September 2021 who were evaluated using the DECAS and ASSET Scales. Our results showed that the agreeableness and emotional stability dimensions of personality, according to the Big Five model, were statistically associated with work relationships (A *p* < 0.0001; ES *p* = 0.0005), work-life balance (A *p* = 0.008; ES *p* = 0.01), overload (A *p* = 0.01; ES *p* = 0.001), job security (A *p* < 0.0001; ES *p* = 0.002), job control (A *p* = 0.001; ES *p* = 0.009), resources and communication (A *p* = 0.0002; ES *p* < 0.0001), and job conditions (A *p* = 0.005; ES *p* = 0.03). The conscientiousness dimension was statistically associated with job control (*p* = 0.02). Doctors from different specialties experienced stress differently, with psychiatrists and doctors from preclinical specialties reporting the lowest levels of stress. Internists and surgeons reported higher levels of stress. This study showed that the dimensions of agreeableness and emotional stability were both associated with variables indicative of the level of stress felt at work.

## 1. Introduction

Physicians are constantly exposed to occupational stress by the nature of their profession [1]. Several studies concerning the mental health of physicians have shown that they are exposed to several stressors, including short time allocated per consultation, overload, long-term job insecurity, low social support at work, and insecurity related to patients’ health conditions and patient suffering [2,3]. These stressors can lead over time to high levels of stress which can then lead to burnout [4]. With increased levels of occupational stress, the performance of doctors can be affected [5]. The performance of doctors is important given the impact that the profession has on humanity [6]. For improved performance, both the quality of doctors’ work and their productivity in the institutions they work in are important [7]. Continuous exposure to stressors in the workplace, in addition to those listed above, such as challenging work shifts, overload, poor communication with superiors and lack of continuing education of doctors can lead to mental and physical exhaustion [8].

Work-life balance can be defined as the balance between work-related responsibilities and personal life [9]. If there is a balance between personal and professional life, the institution to which the employee belongs gains advantages over other institutions in terms of retention of staff and recruitment of new employees [10]. Indicators of balance between personal and professional life include job satisfaction, increased self-esteem and a general feeling of harmony in life [11,12,13].

Job insecurity is perceived as a threat to the stability and continuity of work for an employee. The term job insecurity encompasses both a quantitative aspect, which relates to threats to the workplace as a whole, and a qualitative aspect that refers to the specific tasks of employees [14]. Changes in economic conditions, technology, society and politics can lead to job insecurity [15]. Often, to ensure their standard of living, but also to ensure a certain level of security, a doctor may look for alternatives to their current job, which can result in overload affecting work-life balance [16]. Job insecurity directly affects psychological well-being but can also impact daily performance. It may also lead to decrease in life satisfaction and increased frequency of psychosomatic symptoms [17].

Job control is defined as control of the work process, which refers both to the ability of an employee to decide when and how a job is done, but also to the possession of the skills and initiative necessary to perform the job [18]. Employees who have control over their work schedule, are married and have children have been found to report a good work-life balance and increased job satisfaction [19].

Overload in the professional role is felt by employees where there is the requirement to complete a large volume of work within a limited period [20]. Overload is negatively associated with performance; one reason is that a doctor will devote time to coping with the anxiety and stress generated by the high workload and managing the frustrations generated by the overload [21]. Previous studies have shown that overload is an important predictor of burnout [22]. For this reason, it is important that responsibilities are divided equally among the employees of a clinic to maintain a balance of work demands [23].

Not everyone can get along well with all colleagues; this depends on subjective well-being but also on the ability to tolerate conflict in general [24]. Given the nature of hierarchical structures, relationships between colleagues may differ. For example, the relationship between an attending physician and a resident doctor can be influenced by the superior role of attending physicians, but also by the fact that they are responsible for teaching and supervising resident doctors [25]. The relationship between medical colleagues can be affected by competitiveness, by the inability to ask for an opinion in cases of more difficult pathology of patients, differences in therapeutic approach, but also by personality differences that can create problems for interaction between colleagues. Improving teamwork can contribute to the better management of problems that occur during the treatment of a patient [26]. Good working relationships can positively influence doctors’ well-being [27].

Stress at work can occur due to insufficient resources for the physician to carry out their responsibilities properly [28], but also due to poor communication between physicians and the institution where they work [29]. Good communication can ensure improved job security, increased patient and family satisfaction, shortened hospitalization and encourages collaboration and teamwork, thus preventing future errors [30,31,32].

It is important that the work environment is suitable for carrying out medical tasks in an optimal way. Job conditions refers to the work environment with respect to the physical working conditions, the type of tasks assigned, and can include factors affecting a doctor’s job satisfaction [33]. Psychosocial threats are generated by the organization as a result of poor workplace management with negative effects on the physical and mental health of doctors [34]. In specialties with an increased risk of violence, such as psychiatry or emergency medicine, the level of stress may be higher [35,36].

Stress among physicians depends on the medical specialty practiced, the institution where they work and the resources provided by the institution, but also on the security of the job, the level of control a physician has over their work, the working schedule, the nature of collegiate relationships at work, and on the balance between work and personal life [37,38]. In addition to the factors listed above, the personality makeup of the doctor also plays an important role [39]. A doctor who is emotionally stable and agreeable in interaction is likely to better manage these challenges of professional life [40]. Empathy, modesty, altruism and compassion are personality traits that play a major role in medical practice [41]. The doctor, more than any other professional, must achieve a level of maturity that allows them to harmonize self-knowledge with professional development [42].

There are several theories that have been proposed relating to stress generated at work. These include: (1) person-environment-fit theory, which suggests that personal skills and values must be consistent with the environment in which the subject works in relation to job requirements and opportunities [43]; (2) the demand-control-support model developed in 1979 by Karasek, which argues that an employee‘s subjective well-being is influenced by job characteristics, including job requirements, decision-making and social support at work [44]; (3) equity theory, which suggests that employees who feel unfairly treated at work will be demotivated and stressed [45]; (4) the effort-reward imbalance model, which suggests that the level of stress increases if the effort invested in carrying out tasks at work is not appropriately rewarded [46]; and (5) the transactional model of stress and coping which refers to the way in which an employee adapts and responds to the stress generated by the events that take place at work [47,48].

Informed by the theories referred to above, the purpose of this study was to determine if personality dimensions are associated with the stress felt in various areas of the workplace and, at the same time, to investigate the level of stress felt in these areas by doctors from different specialties, as well as differences between resident and attending physicians. The areas of workplace stress investigated were the following: work relationships, work-life balance, overload, job security, job control, job conditions, access to resources and communication. We hypothesized that emotional stability and agreeableness were the dimensions most associated with different facets of stress at work and that resident physicians would have higher levels of stress than attending physicians. In terms of specialties, we hypothesized that surgeons would have the highest levels of stress in various work-related areas, in contrast to psychiatrists and those in preclinical specialties.

## 2. Materials and Methods

The present research was a cross-sectional study, carried out between February 2021 and September 2021, focusing on doctors who graduated from the Faculty of Medicine, and who carried out their professional activity in Romania. The study was approved by the Ethics Committee of the George Emil Palade University of Medicine, Pharmacy, Science, and Technology of Targu Mureș by decision numbers 1250/28 January 2021 and 1374/20 May 2021. All participants signed an informed consent declaration before being included in the study.

### 2.1. Participants and Procedure

**Figure 1:**
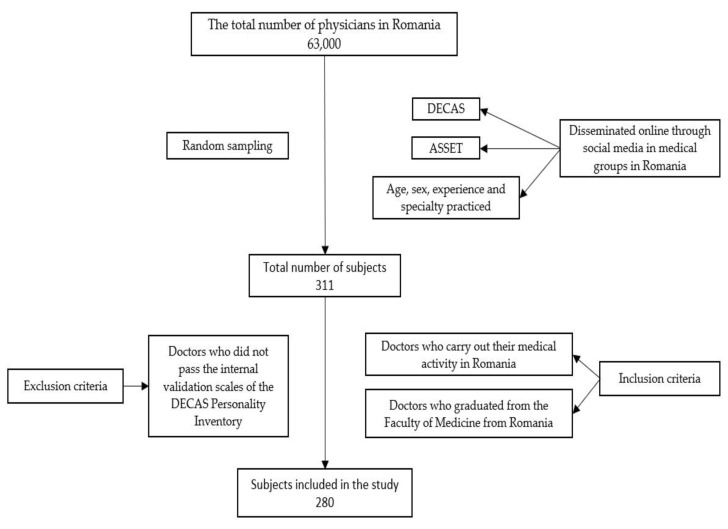
Participants and Procedure.

From a total of 311 subjects initially selected, 280 participants were included in the study. Participants were required to meet the eligibility criteria before being included in the study. The sample subjects were selected by random sampling of physicians from Romania (the population of Romanian physicians is approximately 63,000 [49]). The sample size necessary to fulfill the objectives of this study was calculated using a framework presented in [50] indicating a requirement for 266 participants. The present study sample may be considered representative of the total number of physicians in Romania. From the initial total of 311 subjects, 30 were excluded because they did not pass the internal validation scales of the DECAS Personality Inventory, and one subject was excluded as they were not medically active in Romania. The scales completed by participants included in the study were the DECAS Personality Inventory and, the inventory titled, A Shortened Stress Evaluation Tool (ASSET). In addition to the scales applied in the analysis, the following parameters were recorded: age, sex, experience and specialty practiced. The inclusion criteria applied were: (1) doctors who carried out their medical activity in Romania, and (2) doctors who graduated from the Faculty of Medicine from Romania. An exclusion criterion was doctors who did not pass the internal validation scales of the DECAS Personality Inventory. The questionnaires were disseminated online through social media to medical groups in Romania. The sample selection process is summarized in Figure 1.

### 2.2. Measures

The DECAS Personality Inventory (DECAS) is a local scale developed by Sava et al. for personality assessment according to the Big Five model. It consists of 97 items evaluating five dimensions: openness, extraversion, conscientiousness, agreeableness and emotional stability. The openness dimension emphasizes issues related to culture and intellectual concerns. With respect to the extraversion dimension, people with high levels are sociable, energetic, enthusiastic while those with low scores are calm, passive and less optimistic. The dimension of conscientiousness refers to the desire to assert, the need for order and structure, prudence and responsibility. The agreeableness dimension has the greatest impact on interpersonal relationships. People scoring highly on the dimension are oriented to the needs of others, value harmony, have increased ability to maintain interpersonal relationships; people with low scores on the dimension are competitive, argumentative, authoritative, tough and are concerned with high standards. People with high emotional stability have good self-control, with good management of emotions in conflict situations; emotionally unstable people display irrational reactions, are conflictual, and tend to overestimate the difficulties they encounter. The DECAS Personality Inventory includes three validation scales to avoid distorted answers: (1) social desirability (SD)—measuring the tendency of subjects to answer questions in a way that casts them in a good light, (2) random answers (RD)—subjects give random answers, (3) approving answers (AP)—subjects tend to provide either “true” or “false” responses. The scoring of the scale is interpreted as follows: 20–35: very low values, 35–45: low values, 45–55: average values, 55–65: high values, 65–80: very high values. The Cronbach’s alpha coefficient for the five dimensions varies between 0.70 for the conscientiousness dimension and 0.75 for emotional stability, based on a sample of 1552 Romanian participants [51].

A Shortened Stress Evaluation Tool (ASSET) is a scale developed by Cooper and Cartwright that can evaluate employee’s reactions in different areas. The scale was validated on the Romanian population and is based on the following model: (1) measuring workplace stress factors through: work relationships, work-life balance, overload, job security, job control, access to resources and communication and job conditions; (2) defining the perception of the degree of involvement/employment in both directions by investigating the organization-employee relationship; (3) the effects that the employee’s stress have psychological well-being and physical health; and (4) aspects related to the job, including job satisfaction and the physical conditions of the job. The tool consists of 63 items scored on a 6-point Likert scale and 37 items collecting biographical data. The variables from the tool used in the present study were: work relationships, work-life balance, overload, job security, job control, resources and communication and job conditions. Work relationships refers to interactions between employees at work and evaluates their quality because stressful, tense or difficult relationships will increase the level of stress. Work-life balance relates to how work and personal life combine and whether the inability to maintain this balance generates additional stress. Overload assesses overload and whether this is a source of stress. Job security relates to whether the employee is sure of their place in the organization and whether they consider job changes to be a source of stress. The job control subscale measures the degree to which lack of control at work is perceived as a source of stress. Resources and communication refer to whether the employee feels that they have the necessary training, equipment and resources to carry out their daily activities. Job conditions refers to potential sources of stress related to the nature of work, such as the physical working conditions, the type of tasks, but also the level of job satisfaction. In the present study, low scores on the subscales indicate low levels of stress. The scores are interpreted as follows: <3—very low level, <4—low level, 4–7—medium level, >7 high level, >8 very high level. The internal consistency, as measured by Cronbach’s alpha coefficient, based on the Romanian population, was 0.73, indicating a high level of internal consistency [33,52].

### 2.3. Statistical Analysis

Statistical analysis was performed using GraphPad Prism 9 software. The statistical significance threshold α was set at 0.05. The statistical analysis included descriptive statistics (mean, median, standard deviation), confidence interval CI = 95%, and inferential statistics. To determine the normality of the data distributions, the Kolmogorov–Smirnov test was used. To determine if there were statistically significant differences between the medians of the variables work-relationship, work-life balance, overload, job security, job control, resources and communication and job conditions between resident physicians and attending physicians, the Mann–Whitney test for unpaired data was applied. The Chi^2^ test, Chi^2^ test with Yate’s correction and the Fisher exact test were used to measure the strength of associations between the ASSET variables and dimensions of personality.

## 3. Results

The study included 280 subjects who met the eligibility criteria. The initial number of subjects was 311. Of the 280 subjects evaluated, 233 (83.21%) were women and 47 (16.79%) were men. The mean age was 28.81 ± 4.79 years. Regarding the level of experience, 247 (88.21%) were resident doctors and 33 (11.78%) were attending physicians. The number of subjects according to specialty was as follows: medical specialties, 161 (57.52%), surgical specialties, 39 (13.92%), preclinical specialties, 50 (17.85%) and psychiatry, 30 (10.71%). The demographic characteristics are presented in Table 1.

The descriptive statistics show that the level of all variables indicate stress felt by doctors in all specialties was average according to the scale scores. Our study indicated that there was a statistically significant difference in the stress felt in relation to workplace relationships between internists (5.52 ± 2.03, *p* = 0.0006), surgeons (5.85 ± 2.12, *p* = 0.0005) and psychiatrists (4.5 ± 1.33). Psychiatrists had the lowest stress level. The stress felt associated with work-life balance differed depending on the specialty: internists (5.47 ± 2.10) were more stressed than those in preclinical specialties (4.64 ± 2.23, *p* = 0.01) and psychiatrists (4.17 ± 1.84, *p* = 0.0009); surgeons (6.18 ± 2.15) reported a higher level of stress compared to doctors in preclinical specialties (4.64 ± 2.23, *p* = 0.003) and psychiatrists (4.17 ± 1.84, *p* = 0.0002). Regarding overload, internists (5.33 ± 2.18) reported experiencing stress related to overload more intensely than doctors in preclinical specialties (4.24 ± 1.99, *p* = 0.002) and psychiatrists (3.87 ± 1.57, *p* = 0.0004); surgeons (4.82 ± 2.02) felt the burden more intensely than psychiatrists (3.87 ± 1.57, *p* = 0.03). Surgeons (6.03 ± 1.89) reported the highest levels of stress in terms of the safety of their position at work compared to psychiatrists (4.73 ± 1.93, *p* = 0.01), who reported the lowest level of stress. Regarding job control, there were no statistically significant differences between specialties. Internists (5.53 ± 2.11) reported the greatest stress related to the resources offered but also to communication with superiors; at the other extreme were doctors from the preclinical specialties (4.58 ± 2.07, *p* = 0.01). Physicians in preclinical specialties (4.02 ± 1.13) were more satisfied with working conditions compared to internists (5.11 ± 1.58, *p* < 0.0001), surgeons (4.82 ± 1.19, *p* = 0.001) and psychiatrists (5.63 ± 1.27, *p* < 0.0001). Surgeons (4.82 ± 1.19) were more satisfied than psychiatrists (5.63 ± 1.27, *p* = 0.007). These data are summarized in Table 2 and Table 3.

With respect to level of experience, the stress felt by resident doctors regarding job security (5.71 ± 2.09; 3.79 ± 2.12, *p* < 0.0001) and job control (5.13 ± 2.23; 4.27 ± 2.24, *p* = 0.01) was higher than for attending physicians. The data regarding the level of experience of physicians can be found in Table 4. 

We found that male physicians reported a higher level of stress than women with respect to work-life balance (5.13 ± 2.20; 5.89 ± 1.92, *p* = 0.02). No other statistical differences were observed regarding the other variables. The data mentioned above are presented in Table 5.

To interpret the results and perform statistical tests, we set a value of 45 as a criterion because it represents the threshold between medium and low value. Our study highlighted strong negative associations between the agreeableness dimension and work-relationships (OR = 0.26, *p* < 0.0001), work-life balance (OR = 0.42, *p* = 0.008), overload (OR = 0.39, *p* = 0.01), job security (OR = 0.23, *p* < 0.0001), job control (OR = 0.36, *p* = 0.001), resources and communication (OR = 0.32, *p* =0.0002) and job conditions (OR = 0.22, *p* = 0.005). The emotional stability dimension had statistically significant negative associations with work-relationships (OR = 0.33, *p* = 0.0005), work-life balance (OR = 0.44, *p* = 0.01), overload (OR = 0.30, *p* = 0.001), job security (OR = 0.36, *p* = 0.002), job control (OR = 0.42, *p* = 0.009), resources and communication (OR = 0.27, *p* < 0.0001) and job conditions (OR = 0.28, *p* = 0.03). The conscientiousness dimension was associated only with the job control variable (OR = 0.48, *p* = 0.02). All the above associations are presented in Table 6.

## 4. Discussion

Provision of medical services often involves high levels of stress, being the field with the highest level, as doctors are responsible for the lives of patients and mistakes, that may be due to overload or fatigue, can often be irreversible [53]. For this reason, it is important for doctors to be in good physical and mental condition for patients to receive the best care [54]. The results obtained indicate that the level of stress felt by doctors in Romania is medium, which may be attributed to the development of coping skills with increasing experience. It is important to note that the doctors included in our study undertook their medical practice in the public healthcare system, where it has been suggested by other researchers that stress levels are lower than in the private healthcare system [55]. This may be because, in the private healthcare system, the demands of both patients and relatives, and of the employer, increase compared to the public healthcare system, but also to job insecurity which does not exist in the public sector [55]. The levels of stress vary depending on the specialty practiced—relationships at work in psychiatric wards are better than those in the surgical and internal medicine wards. Relationships between colleagues are based on mutual emotional support, support for professional development and mutual help related to daily tasks [56,57]. Psychiatrists communicate more clearly and interact more easily emotionally due to the central role that these aspects play in daily practice with patients [25]. With respect to stress felt due to work-life imbalance, surgeons and interns reported that responsibilities at work affected their family life, an observation confirmed by Tait et al. [58]. Psychiatrists may manage these things better due to the nature of the specialty and the ability to apply techniques acquired throughout the career to control emotions and behavior and to delimit personal life from professional life [59]. As for psychiatrists, those in preclinical specialties appeared to be better able to manage work-life balance, most likely because they can control the number of procedures they perform, the duration of procedures and the way they examine hospitalized patients and consult in ambulatory services [60]. Moreover, doctors in preclinical specialties are not affected by the development of the response to treatment of the hospitalized patient or, in extreme cases, their death, because they do not develop a doctor-patient relationship [61]. A study by Ganeshan et al. showed that radiologists who were in academic roles failed to balance work and personal life due to the multitude of activities they performed [62]. The stress generated by overload was felt more strongly by surgeons and internists, which also influences the work-life balance, due to the large number of patients with multiple comorbidities that must be managed and investigated over a relatively short period of hospitalization, and, in the case of surgeons, the long time spent in the operating room [63]. Psychiatrists reported the lowest levels of stress related to job security; this may be because this specialty can be practiced in the private sector, and does not require substantial investment, a therapeutic team or special conditions, as is required in surgical specialties [64,65]. With respect to job satisfaction, our study highlighted that psychiatrists were the most satisfied with the environment in which they worked, in contrast to doctors from preclinical specialties. This can be explained by the fact that the interaction between physicians and patients and their relatives is important for psychological well-being [66]. Most patients do not remember the name of the radiologist who consulted them, which can be explained by the increased level of anxiety they feel during the consultation, but also as they tend to only experience one consultation [67], an issue that contributes negatively to the satisfaction of the radiologist’s job. With increasing experience, the satisfaction among psychiatrists differs, being conditioned by the socio-cultural interactions, but also by the environment in which they carry out their activity [68]. A doctor’s job satisfaction can be increased by developing the skills to work in a team, by positive individual relationships with colleagues, and by individual progress in work, but also by improving interactions with patients [69].

Our results indicate that resident doctors have less control over the work they do compared to attending physicians because they do not have sufficient experience, and that they perceive the workplace as an additional source of stress because they have little control over the work performed. Resident doctors consider that medical activities are strongly influenced by the workplace environment, as well as by the attending physician with whom they carry out their role [55]. Resident doctors have few possibilities to influence treatment [70]. Due to low autonomy in medical practice among young doctors, job insecurity is increased [71]. Our study indicates that long-term job security is low among resident physicians, with a permanent fear of losing their job, generating additional stress. This is because, in Romania, after completing residency and obtaining the title of fellow or attending physician, doctors must find a new job by themselves because they are employed on fixed-term contracts. At the beginning of their career, resident doctors experience a high level of job insecurity [72]. Job insecurity impacts the psychological well-being of medical staff as a result of multiple factors, including temporary and unstable work, frequent changes in the work environment and unfavorable working conditions (overload, increased number of working hours), with consequences for physical and mental health [73].

With respect to the level of stress associated with maintenance of a suitable work-life balance, male participants reported a higher level. In the literature, there are contradictory results, with some studies indicating that men have a better work-life balance than women, or that there is no difference [74,75,76]. Shanafelt et al. demonstrated that, over time, there has been a decrease in satisfaction with work-life balance among doctors [77]. Finding a suitable work-life balance can be difficult for men due to the desire to succeed at work, as there is a view that men must financially support the family [78]. As a result, there may be a conflict between the time allocated to family and work [79]. This can generate additional stress over time in seeking to achieve work-life balance; contact with family members has been shown help balance professional life [80].

According to the results obtained, both the emotional stability dimension and the agreeableness dimension are associated with the stress felt in all the areas considered. Our results have been confirmed by other researchers for the general population [81,82]. Physicians with increased agreeableness will be able to adapt to the situation more easily by finding opportunities, but also solutions to problems related to both work and personal life [83]. Doctors who have low emotional stability will have less time available for the family because they will worry and focus on negative events [83].

Increased emotional stability reduces stress felt in terms of keeping the current job, as does increased agreeableness. Increased neuroticism, but low agreeableness, maintains anxiety and fear and consequently increases job insecurity [51,84]. Wu et al. using data from the HILDA Survey, found that there was an association between agreeableness, emotional stability and job insecurity, and that these personality traits decreased due to job insecurity over the long term in the general population [85]. A study carried out by Griep et al. on a Flemish population sample who were employed on fixed-term contracts across all sectors, including the medical field, found that there was a direct relationship between the perception of job insecurity and mental health [86].

The results of our study show that low emotional stability and low agreeableness are associated with the perception of low control over work. Physicians with increased neuroticism are more likely to develop anxiety if they fail to control the work they do [87]. Wu et al. showed that agreeableness increases in the long term for doctors who manage to control their schedules and work [88]. The dimension of conscientiousness was found to be positively associated with job control. A doctor who is organized, punctual, ambitious, persevering and strives to achieve the objectives manages their work better, and the environment in which they work, and also manages to better organize their schedule [88].

Both resources and communications and job conditions are closely linked to the environment in which a doctor works. Job conditions are related to job satisfaction. Both agreeability and emotional stability are positively associated with positive perceptions of resources and communication and job conditions. An agreeable person is characterized by empathy, compassion, sympathy and easy communication [51], attributes that influence both patients and colleagues trust in them; this explains why job satisfaction increases—because the environment in which they work is a pleasant one [89]. Low stability or increased neuroticism leads to anxiety, fear, irritability, anger, and frustration [90], with the inability to control feelings [91], resulting in poor management of personal emotions and of patients in a stressful work environment [92].

## 5. Limitations

There are several limitations of the study that should be addressed in future work. The first is that the study is of a cross-sectional type. Stress levels may be different depending on daily events, with these being evaluated only once for each participant. Future research is needed in which the level of stress is investigated over several stages. A second limitation is that the questionnaires were self-administered; there may be a risk that the answers were distorted in the case of the ASSET questionnaire. The DECAS personality inventory includes internal validation scales to enable checking for distorted responses. Although the sample is representative of doctors in Romania, future research on a larger sample should be considered. Considering that an increased level of stress can lead to burnout syndrome, in the future, research should be carried out to evaluate the association between stress-generating factors and burnout among doctors from different specialties.

## 6. Conclusions

This study confirmed that the dimensions of agreeableness and emotional stability were both associated with variables that indicates the level of stress felt at work, both in terms of relationships and the work itself. With respect to the level of stress, resident physicians reported a higher level than attending physicians. Male physicians reported higher levels of stress regarding work-life balance. Doctors from different fields experienced varying degrees of stress. Surgeons reported the highest levels of stress generated by work relationships, work-life imbalance and job insecurity. Internists felt more strongly the stress generated by overload, the control they have over the work performed, the schedule and the tasks, but also the resources needed for patient care and communication with superiors. Psychiatrists reported the highest levels of stress regarding job conditions. The provision of programs in hospitals which doctors can use to develop self-awareness and learn strategies to address certain areas of weakness, should increase the quality of medical care and increase the satisfaction of both patients and doctors.

## Figures and Tables

**Table 1:** Demographic characteristics of the sample.

Sample Characteristics	n = 280
Gender, n (%)	
Female	233 (83.21)
Male	47 (16.79)
Age range,	25–58
M (SD)	28.81 (4.79)
Experience, n (%)	
Attending	33 (11.78)
Resident	247 (88.21)
Specialty, n (%)	
Medical	161 (57.52)
Surgical	39 (13.92)
Preclinical	50 (17.85)
Psychiatry	30 (10.71)

Legend: M = mean; SD = standard deviation.

**Table 2:** Mean values of variables related to stress within different medical specialties.

	Internists n = 161			Surgeons n = 39			Preclinicals n = 50			Psychiatrists n = 30		
	M	SD	95% CI	M	SD	95% CI	M	SD	95% CI	M	SD	95% CI
Work relationships	5.52	2.03	5.20 to 5.83	5.85	2.12	5.16 to 6.53	5.04	2.12	4.44 to 5.64	4.5	1.33	4.00 to 4.99
Work-life balance	5.47	2.10	5.14 to 5.80	6.18	2.15	5.48 to 6.88	4.64	2.23	3.99 to 5.28	4.17	1.84	3.48 to 4.85
Overload	5.33	2.18	4.99 to 5.66	4.82	2.02	4.16 to 5.48	4.24	1.99	3.68 to 4.80	3.87	1.57	3.28 to 4.45
Job security	5.57	2.24	5.21 to 5.91	6.03	1.89	5.41 to 6.64	5.26	2.28	4.61 to 5.91	4.73	1.93	4.01 to 5.45
Job control	5.24	2.32	4.87 to 5.59	5.15	2.15	4.46 to 5.85	4.54	1.96	3.98 to 5.09	4.6	2.24	3.76 to 5.44
Resources and communication	5.53	2.11	5.19 to 5.85	5.46	2.14	4.77 to 6.16	4.58	2.07	3.99 to 5.17	5.3	1.77	4.64 to 5.96
Job conditions	5.11	1.58	4.86 to 5.35	4.82	1.19	4.44 to 5.21	4.02	1.13	3.69 to 4.34	5.63	1.27	5.16 to 6.11

Legend: M = mean; SD = standard deviation; CI = confidence interval.

**Table 3:** Comparations between specialties regarding the level of stress.

		Internists n = 161		Surgeons n = 39		Preclinicals n = 50		Psychiatrists n = 30	
		U	*p* *	U	*p* *	U	*p* *	U	*p* *
Work relationships	Internists n = 161			2820	0.32	3376	0.08	1167	0.006
	Surgeons n = 39	2820	0.32			741	0.05	358	0.005
	Preclinicals n = 50	3376	0.08	741	0.05			683.5	0.503
	Psychiatrists n = 30	1167	0.006	358	0.005	683.5	0.5		
Work-life balance	Internists n = 161			2658	0.13	3113	0.01	1506.5	0.0009
	Surgeons n = 39	2658	0.13			620.5	0.003	279	0.0002
	Preclinicals n = 50	3113	0.01	620.5	0.003			661.5	0.37
	Psychiatrists n = 30	1506.5	0.0009	279	0.0002	661.5	0.37		
Overload	Internists n = 161			2705.5	0.17	2907	0.002	1433	0.0004
	Surgeons n = 39	2705.5	0.176			825.5	0.21	409.5	0.03
	Preclinicals n = 50	2907	0.002	825.5	0.21			680	0.48
	Psychiatrists n = 30	1433	0.0004	409.5	0.03	680	0.48		
Job security	Internists n = 161			2728.5	0.2	3737.5	0.44	1922.5	0.07
	Surgeons n = 39	2728.5	0.20			776.5	0.09	388	0.01
	Preclinicals n = 50	3737.5	0.44	776.5	0.09			657.5	0.35
	Psychiatrists n = 30	1922.5	0.07	388	0.01	657.5	0.35		
Control	Internists n = 161			3124.5	0.96	3361.5	0.07	2039	0.17
	Surgeons n = 39	3124.5	0.96			797	0.13	474.5	0.17
	Preclinicals n = 50	3361.5	0.07	797	0.13			749	0.99
	Psychiatrists n = 30	2039	0.17	474.5	0.17	749	0.99		
Resources and communication	Internists n = 161			3092.5	0.88	305	0.01	2304.5	0.68
	Surgeons n = 39	3092.5	0.88			762.5	0.07	572	0.87
	Preclinicals n = 50	305	0.01	762.5	0.07			588	0.10
	Psychiatrists n = 30	2304.5	0.68	572	0.87	588	0.10		
Job conditions	Internists n = 161			2818.5	0.31	2366.5	<0.0001	1891.5	0.05
	Surgeons n = 39	2818.5	0.31			592	0.001	369.5	0.007
	Preclinicals n = 50	2366.5	<0.0001	592	0.001			264	<0.0001
	Psychiatrists n = 30	1891.5	0.05	369.5	0.007	264	<0.0001		

Legend: * Mann–Whitney test, *p* < 0.05 (two-tailed).

**Table 4:** Comparison between physicians regarding the level of experience.

	Resident Physicians n = 247 M ± SD (Median)	Attending Physicians n = 33 M ± SD (Median)	*p* Value *
Work relationships	5.40 ± 2.07 (5.00)	4.97 ± 2.19 (5.00)	0.15
Work-life balance	5.22 ± 2.12 (5.00)	5.58 ± 2.57 (6.00)	0.46
Overload	4.93 ± 2.12 (5.00)	4.67 ± 2.22 (4.00)	0.41
Job security	5.71 ± 2.09 (6.00)	3.79 ± 2.12 (4.00)	<0.0001
Control	5.13 ± 2.23 (5.00)	4.27 ± 2.24 (4.00)	0.01
Resources and communication	5.39 ± 2.11 (5.00)	4.79 ± 1.99 (5.00)	0.09
Job conditions	4.96 ± 1.48 (5.00)	4.69 ± 1.63 (4.00)	0.18

Legend: M = mean; SD = standard deviation; * Mann–Whitney test, *p* < 0.05 (two-tailed).

**Table 5:** Gender comparison regarding the level of stress.

	Female n = 233 M ± SD (Median)	Male n = 47 M ± SD (Median)	*p* Value *
Work relationships	5.53 ± 2.00 (6.00)	5.32 ± 2.03 (5.00)	0.47
Work-life balance	5.13 ± 2.20 (5.00)	5.89 ± 1.92 (6.00)	0.02
Overload	4.81 ± 2.12 (4.00)	5.38 ± 2.09 (5.00)	0.06
Job security	5.55 ± 2.20 (6.00)	5.14 ± 2.06 (5.00)	0.21
Control	5.08 ± 2.28 (5.00)	4.78 ± 2.01 (4.00)	0.33
Resources and communication	5.27 ± 2.14 (5.00)	5.57 ± 1.82 (5.00)	0.42
Job conditions	4.95 ± 1.54 (5.00)	4.80 ± 1.26 (4.00)	0.76

Legend: M = mean; SD = standard deviation; * Mann–Whitney test, *p* < 0.05 (two-tailed).

**Table 6:** Associations between personality dimensions and variables related to occupational stress.

	Openness			Extroversion			Conscientiousness			Agreeableness			Emotional Stability		
	OR	95% CI	*p*	OR	95% CI	*p*	OR	95% CI	*p*	OR	95% CI	*p*	OR	95% CI	*p*
Work relationships	1.08	0.53 to 2.16	0.82 *	1.16	0.66 to 2.07	0.62 *	1.05	0.57 to 1.97	0.86 *	0.26	0.14 to 0.49	<0.0001 *	0.33	0.18 to 0.64	0.0005 *
Work-life balance	1.64	0.74 to 3.51	0.31 **	1.55	0.83 to 2.88	0.17 *	1.01	0.54 to 1.88	0.98 *	0.42	0.22 to 0.80	0.008 *	0.44	0.23 to 0.81	0.01 *
Overload	1.05	0.47 to 2.30	0.91 *	1.16	0.58 to 2.31	0.68 *	1.56	0.72 to 3.46	0.25 *	0.39	0.19 to 0.85	0.01 *	0.30	0.15 to 0.67	0.001 *
Job security	1.22	0.59 to 2.50	0.59 *	0.90	0.48 to 1.69	0.74 *	0.77	0.41 to 1.46	0.41 *	0.23	0.12 to 0.46	<0.0001 *	0.36	0.19 to 0.69	0.002 *
Job control	1.29	0.59 to 2.91	0.64 **	0.77	0.41 to 1.48	0.42 *	0.48	0.25 to 0.92	0.02 *	0.36	0.19 to 0.69	0.001 *	0.42	0.22 to 0.81	0.009 *
Resources and communication	0.71	0.37 to 1.36	0.30 *	0.93	0.51 to 1.72	0.82 *	0.93	0.51 to 1.73	0.82 *	0.32	0.17 to 0.59	0.0002 *	0.27	0.14 to 0.53	<0.0001 *
Job conditions	0.62	0.22 to 1.69	0.37 *	1.01	0.35 to 2.65	0.79 **	0.60	0.23 to 1.73	0.48 **	0.22	0.07 to 0.61	0.005 **	0.28	0.09 to 0.83	0.03 **

Legend: OR = odds ratio; CI = confidence interval; * = Chi ^2^ test, *p* < 0.05 (two-tailed); ** = Chi ^2^ test with Yate’s correction, *p* < 0.05 (two-tailed).

## Data Availability

Not applicable.

## Abbreviations

DECAS	DECAS Personality Inventory
O	Openness dimension
E	Extraversion dimension
C	Conscientiousness dimension
A	Agreeableness dimension
ES	Emotional stability dimension
ASSET	A Shortened Stress Evaluation Tool
